# The Benefits and Challenges of Managing Asthma in Hispanic Families in South Texas: A Mixed-Methods Study

**DOI:** 10.3389/fpubh.2017.00150

**Published:** 2017-06-30

**Authors:** Genny Carrillo, Maria J. Perez-Patron, Rose L. Lucio, Lucia Cabrera, Alyssa Trevino, Xiaohui Xu, Nelda Mier

**Affiliations:** ^1^Department of Environmental and Occupational Health, School of Public Health, Texas A&M University, College Station, TX, United States; ^2^Department of Epidemiology and Biostatistics, School of Public Health, Texas A&M University, College Station, TX, United States; ^3^McAllen Campus of the Texas A&M University School of Public Health, McAllen, TX, United States

**Keywords:** focus groups, asthma education, asthma management, quality of life, Hispanic families, Children’s Health Survey for Asthma, US–Mexico border

## Abstract

**Background:**

Understanding the experience of Hispanic parents of children diagnosed with asthma can be useful in the delivery of effective and meaningful asthma education. In order to assess the needs of Hispanic families with asthmatic children in South Texas, investigators utilized a combination of qualitative and quantitative research methods.

**Objectives:**

This study aimed (1) to assess the impact of asthma in the quality of life of Hispanic children and their families and (2) to identify barriers and challenges to asthma management as perceived by parents of children diagnosed with asthma.

**Methods:**

A mixed-methods study included a quality-of-life survey and focus group discussions. The Children’s Health Survey for Asthma (CHSA) was completed by 90 parents of children with asthma. Three focus groups were conducted with 15 low-income, Hispanic parents of asthmatic children to assess their needs and experience in managing the disease.

**Results:**

Results from the CHSA showed that asthma significantly affects the quality of life of children with asthma and their families, particularly the emotional dimensions and the child’s physical health. Fifty-three percent of the children had visited the emergency room, and 51% had been hospitalized due to asthma. One out of five parents had missed work, and 27% of children had missed school in the past 2 weeks due to the child’s asthma. In the focus group discussions, the key themes emerging included lack of asthma knowledge, the burden of disease for asthmatic children and their families, and the importance of asthma education and self-management behaviors for asthma control.

**Conclusion:**

One of the main challenges faced by Hispanic families with asthmatic children is the lack of asthma-related knowledge to help understand and control their children’s disease. Lack of knowledge and self-management skills lead to significant stress and anxiety among children with asthma and their parents. Results highlight that while asthma has an effect on the quality of life of children and their families, particularly on the emotional health domain, a wide dissemination of asthma management education in different settings might help prevent asthma attacks and improve symptom control among those suffering from this disease along the US–Mexico border.

## Introduction

Asthma is the most common chronic disease among children in the United States. It is estimated that asthma affects 25.6 million people in the United States, including 6.8 million children under the age of 18 (1). Between 2001 and 2010, asthma prevalence in children increased at a rate of 1.5% per year to reach a prevalence rate of 8.4% (2). Uncontrolled asthma greatly affects the quality of life of individuals and their families. An average of 4.5 days per year of school or work are missed due to an asthma attack (3), and approximately 50% of children diagnosed with asthma miss at least 1 day of school due to an asthma attack (4). Research indicates that children with asthma have higher rates of obesity, BMI, and emotional symptoms linked to stress. Parents of asthmatic children perceive that asthma affects children’s physical activity levels, consequently leading them to a sedentary lifestyle (5). In 2014, current asthma was found in 6.7% of Hispanic children under 18 years of age and 7.1% of Mexican–American children (6, 7). Asthma is also a chronic disease with high economic costs. In many cases, individuals suffering an asthma attack must go to an emergency room or be hospitalized. According to the CDC, in 2014, 48% of children younger than 18 years old with current asthma reported having one or more asthma attacks (6). The number of asthma patients under the age of 18 discharged from hospitals was reported as 18.3 per 10,000 patients in 2010 (8). In the past decade, health-care expenditures due to asthma increased from $53 billion to $56 billion (9).

Children living along the US–Mexico border region are significantly affected by asthma. The illness accounts for the leading cause of hospitalizations among border children (10), and research shows higher rates of asthma and allergic disease on the US side of the border (11). Hispanic families, primarily those who are considered low income, are more likely to have been diagnosed with asthma than their counterparts in a higher socioeconomic status. Low-income families are also more likely to live in apartments and other domiciles in neighborhoods where environmental asthma triggers are numerous. Air quality is a particular concern in the border region due to many environmental conditions such as: high exposure rates of pesticides, the approximate 3,000 factories located along the border emitting air pollutants, shortages of waste disposal, and water pollution. Health problems in the border region are also aggravated due to poor access to health insurance, health-care providers, and cultural beliefs about medical care (12, 13). An estimated 7.3% of adults and 9.1% of children had asthma in Texas. This translates into more than 1.4 million adults and 617,000 children with asthma. In 2013, there were over 201,000 asthma Medicaid claims reported among adults and over 635,000 asthma Medicaid claims among children. The Asthma Medicaid expenditures totaled $28.7 million among adults and $91.9 million among children.

Although asthma is a lifelong disease and there is no cure for it, research shows that asthma symptoms and attacks can be controlled and prevented by avoiding environmental triggers and by adhering to medication treatments. A Canadian study found that high exposure levels to indoor and outdoor environmental contaminants triggered asthma attacks and increased the risk of emergency room visits and hospital admissions for children with asthma (14). Other studies found that chronic exposure to indoor allergens, such as mold, pet dander, rats and other rodents, cockroaches, and dust mites are associated with asthma (15). Inhaled corticosteroid medication, when used regularly, has been shown to improve asthma control and to reduce the risk of asthma emergency department visits and hospitalizations in multiple large-scale observational and randomized controlled clinical trials (16–18).

Investigating how Hispanic families deal with the care of a child with asthma is of great public health significance. It will shed light on the particular barriers, challenges, and limitations that these families face daily and will help health professionals to develop more effective and culturally competent interventions. Many families face the double jeopardy of socioeconomic and cultural difficulties in their efforts to learn about asthma and self-management skills, due to the lack of medical insurance, their limited knowledge or education related to asthma, and their lack of understanding of programs that are available to help them deal with their asthmatic child’s medical needs and concerns. Learning to manage asthma results in improved quality of life for children and their families and reduces health-care costs. Asthma management programs are effective in reducing the onset and severity of asthma by teaching individuals to identify and manage indoor and outdoor environmental asthma triggers and to adhere more effectively to their medication treatment (15, 19). Despite the evidence that asthma management improves symptoms and decreases hospitalizations and emergency room visits (7, 19, 20), there are many factors that limit participation in these programs. For Hispanic families, common barriers to participation in asthma management programs include poverty, lack of health insurance, difficulty in arranging transportation to physician visits, language barriers, lack of asthma management information for clinical improvement, parents’ concerns about potential long-term complications for their children with asthma, and potential dependency and side effects of medications (21–24).

The purpose of this study was twofold: (1) to assess the impact of asthma in the quality of life of diagnosed children and their families and (2) to identify barriers and challenges to asthma management as perceived by parents of children diagnosed with asthma living in the US–Mexico border region.

## Materials and Methods

This was a mixed-methods study that included a health-related quality-of-life survey and focus group discussions. Patient-centered outcome measures have been developed to assess the burden of a disease from the point of view of the patient. The Children’s Health Survey for Asthma (CHSA) is designed to measure the perceived impact of asthma on the quality of life of children with chronic asthma and their families. Focus groups can be used to complement surveys in several ways. For this study, they were used to provide a more in-depth understanding of the quantitative results while considering the specific characteristics of Hispanic families of children with asthma living in the US–Mexico border region. Both, the survey and the focus group components of the study, were reviewed and approved by the Institutional Review Board at Texas A&M University.

### Data Collection

#### Children’s Health Survey for Asthma

The CHSA is a condition-specific, self-reported, functional measure for parents of children 5–12 years of age with chronic asthma. The CHSA was designed by the American Academy of Pediatrics to measure quality of life for children with asthma in five domains: physical health of the child, child activity, family activity, child emotional health, and family emotional health. The CHSA also includes questions about asthma triggers, health-care utilization, and demographic characteristics (25). The CHSA is copyrighted by the American Academy of Pediatrics © 2000.

The CHSA was administered during the baseline visit of an Asthma and Healthy Homes education intervention in 2016 (7). Ninety parents and guardians of children with asthma completed the CHSA. Recruitment of participants occurred during the spring of 2016 at eight elementary and two middle schools in Weslaco ISD in Hidalgo County, TX, USA, using convenience sampling. Consent forms were sent home to families of students with asthma through the schools’ nurses. Families who returned the signed consent form were invited to participate in an Asthma and Healthy Homes Education Intervention Program. Sixty-eight percent of those who signed the consent form were successfully recruited in the study. Attrition from the program for follow-up visits were primarily attributed to the inability to contact participants due to phone numbers that were either incorrect or disconnected or because the families had moved away from their residence. Less than 5% of those contacted refused to participate in the education program.

#### Focus Groups

In addition to the CHSA data, three focus groups were conducted during the summer of 2016 with fifteen parents of children with asthma. *Promotoras* (community health workers) recruited participants at three community health centers in Hidalgo County, TX, USA. Informed consent, including authorization to audio record and demographic information were obtained as participants arrived at the focus group sites and before starting the discussion. All focus groups were conducted in Spanish following a semi-structured format covering topics related to asthma knowledge and beliefs, burden of disease, self-management behavior, and access to health care and asthma education. Two bilingual members of the research team were present during the focus groups. One was in charge of moderating the discussion based on the questions in the discussion guide (see Table 1), while the other took notes of the focus group dynamic that were subsequently utilized when transcribing the audio recordings of the session. Each focus group lasted about an hour. Compensation for the time of participants was provided *via* a $20 gift card and an allergen mattress and pillow cover for their child.

**Table 1:** Focus group discussion guide.

Topic	Prompting question
Knowledge	In your words, what is asthma?
	Were you familiar with asthma before your child was diagnosed?
Diagnosis	How old was your child when s/he was diagnosed?
	Can you talk about the circumstance leading to the diagnosis?
Severity	How severe is the asthma? Does s/he take any medication?
Asthma effects	How does asthma affect your child’s life? What about your family?
Access to health care	Do you go to a primary doctor or clinic to help you control your child’s asthma?
Asthma control	Are there things that you can do to make the asthma better?
Asthma education	Have you received asthma education in the past?

### Data Analysis

#### Children’s Health Survey for Asthma

Descriptive statistics of the CHSA and focus groups samples were estimated. For the CHSA, normalized mean scale scores were calculated for the five domains of the survey: physical health of the child, child activity, family activity, child emotional health, and family emotional health. Each normalized subscale can range from 0 to 100, with higher values indicating better outcomes. For all subscales, higher scores indicate better health status. Internal consistency, the degree to which all of the subscale items measure the same construct, was assessed with Cronbach’s alpha correlations analyses. All statistical analyses were performed using Stata 14.2 (26).

#### Focus Groups

Descriptive statistics of the focus groups participants were calculated and are displayed in Table 2. Since the focus group sessions were centered on the group discussion, the demographic information available for focus groups participants is more limited than for survey data respondents. Audio recordings of the focus groups were transcribed and translated into English for analysis purposes. Field notes taken during the focus group discussions were also used to supplement the transcripts. Members of the research team who were fluent in English and Spanish analyzed the focus group transcripts. Themes were identified by two of the co-investigators and then reviewed by a third following a purpose-driven framework analysis (27–30). Researchers identified, coded, and analyzed keywords and emerging themes that indicated participants’ perceptions and opinions. In cases of disagreement about keywords or topics during the coding process, the team discussed the issue until reaching a consensus. If no consensus was reached, the principal investigator’s decision prevailed. Questions, conversation fillers, and irrelevant comments were blocked out.

**Table 2:** Demographic characteristics of focus group participants, *n* = 15.

Female	93%
Age, mean (SD)	40.1 (6.8)
Hispanic	100%
Less than high school education	69%
Married or in a union	85%
More than one child with asthma	38%
Age of children with asthma, mean (SD)	11.4 (4.9)
Age of diagnosis, mean (SD)	7.7 (5.1)
Child has CHIP or Medicaid health insurance	100%
Received asthma education	69%

## Results

### Children’s Health Survey for Asthma

Table 3 summarizes the characteristics of the survey respondents and their children. The majority of participants (94%) were female with a mean age of 37.4 (SD = 8.3). All survey participants self-identified as Hispanic. Forty-six percent of participants had less than high school education, and more than half (57%) reported having an annual household income of less than $15,000. Most of their children (85%) had access to health insurance through Medicaid. Less than half of the respondents (37%) reported having received asthma education in the past, and while three-fourths (74%) of the respondents had a family history of allergies, only 47% had a positive family history of asthma. One out of five households interviewed had more than one child with asthma. The mean age of children with asthma was 9.8 years (SD = 3.6) and, on average, children had been diagnosed with asthma at 4 years of age (SD = 3). Almost all the children (97%) were currently taking asthma medication, although only half of them (53%) used it every day, 41% of the respondents worried about the cost of the child’s asthma medication. In the past 2 weeks, 23% of the children and 16% of their parents or guardians had missed school or work due to the child’s asthma. About half of the children had visited an emergency room (53%) or been hospitalized due to asthma (51%).

**Table 3:** Characteristics of the survey respondents and children with asthma, *n* = 90.

Respondent’s gender	
Male	6%
Female	94%
Respondent’s age, mean (SD)	37.4 (8.3)
Hispanic/Latino	100%
Respondent’s relationship to child	
Parent	98%
Grandparent/other relative	2%
Respondent’s marital status	
Married	71%
Separated/divorced	19%
Single/widowed	10%
Respondent’s education	
<High school	46%
High school graduate	24%
>High school	30%
Health insurance	
Private	9%
Public	85%
None/don’t know	6%
Household income	
<15,000	57%
15,000–29,999	33%
30,000+	9%
More than one child with asthma in the household	21%
Received asthma education	37%
Child’s gender	
Male	57%
Female	43%
Child’s age, mean (SD)	9.8 (3.6)
Age of diagnosis, mean (SD)	4.0 (3.0)
Child uses asthma medication	97%
Child uses asthma medication everyday	53%
Worry about the cost of child’s medical care for asthma	41%
Child ever hospitalized for asthma	51%
Child ever in the ER	53%
Missed school in the past 2 weeks	27%
Missed work in the past 2 weeks	20%
Family history	
Asthma in the family?	47%
Allergy in the family?	74%

Table 4 shows the normalized mean scores for each of the five scales of the CHSA. The scores can range from 0 to 100, where higher scores indicate better health status. All five dimensions are significantly affected by the child’s condition. Of the three child’s subscales assessed in the survey, parents reported that the child’s emotional health $\left({\bar{x}}_{\text{CEH}}=70\right)$ was the most affected while the child’s activities $\left({\bar{x}}_{\text{CA}}=79\right)$ seemed to be the least affected. The child’s physical health $\left({\bar{x}}_{\text{CPH}}=74\right)$ fell somewhere in the middle. Similarly, for the family dimensions, emotional health $\left({\bar{x}}_{\text{FEH}}=65\right)$ appeared to be the most affected, scoring even lower than the child’s emotional health. Families’ activities, on the other hand, were the least affected by the child’s asthma $\left({\bar{x}}_{\text{FA}}=88\right)$. All dimensions reported high internal consistency, as measured by Cronbach’s alpha correlations greater than 0.90 (25).

**Table 4:** Scores for the Children’s Health Survey for Asthma (CHSA), *n* = 90.

Subscale	Mean	SD	Min	Max	Cronbach’s alpha
**Child**					
Physical health	74.3	22.1	13.3	100	0.92
Emotional Health	69.7	34.1	0	100	0.94
Activity	79.2	28.8	0	100	0.94
**Family**					
Emotional health	65.3	18.9	17.6	100	0.91
Activity	88.0	21.5	12.5	100	0.91

*Scores range from 0 to 100. Higher scores represent better outcomes*.

### Focus Groups

Fifteen Hispanic parents of children with Asthma participated in the three focus groups (see Table 2). As in the survey, focus group participants were overwhelmingly female (14 out of 15) and slightly older than the survey respondents (mean = 40.1 years; SD = 6.8), probably because their eligibility criteria were not tied to their child’s school grade. The mean age of children with asthma was 11.4 years old (SD = 4.9), older than the children of the survey respondents. Since the recruitment of focus group participants was conducted at community health centers, more parents had received asthma education in the past as compared to survey respondents (69% vs. 37%). Over two-thirds of the focus groups respondents (69%) had less than high school education compared to only 46% of survey respondents, and 85% reported being married or in a cohabiting union. All of the children had health insurance through CHIP or Medicaid. Table 1 shows the questions used to guide the focus group discussions. Key themes emerging from the focus group discussions were: *lack of asthma knowledge* among both parents and children, the *burden of disease* for children and their families, *self-management* behaviors, including trigger identification and treatment compliance, and the importance of *asthma education* for asthma knowledge and asthma control. These themes emerged in all focus groups. Presented below are excerpts of the focus group discussions organized by key identified themes.

#### Lack of Asthma Knowledge

When asked about their experience as parents of children with asthma, the lack of asthma knowledge emerged as a common theme, as well as, a great source of stress in all focus groups. Only one of the participants, who also suffered from asthma, had knowledge of the disease before her child’s diagnosis. All other parents learned about the disease when their children were diagnosed, usually after dealing with multiple respiratory illnesses and complications before a diagnosis was made:

– “I did not know what asthma was for a while. My daughter would start choking and I did not know why, I did not know it was asthma until the doctor told me it was because of the asthma” Focus Group 2 (FG2)
– *“Sometimes you don*’*t know. You see the symptoms but don*’*t know that it is a specific illness” FG2*
– “Yes, at first you think it is the flu, or a cold, or a cough. But it gets worse and it does not go away” FG2
– “No, I did not know anything about asthma before” Focus Group 3 (FG3)
– “Me either. I got scared the first time she fainted” FG3
– “It is scary when you do not know why they are agitated or feeling sick” FG3

#### Burden of Disease

The asthma condition was defined in the way that it was experienced: by its symptoms, of which the most important was having difficulty or not being able to breathe. Other commonly mentioned symptoms included chest pain, tiredness, coughing, and wheezing:

– *“Just whenever is really hot or really cold she had difficulty breathing, she*’*s had this problem since pre-kindergarten” FG2*
– “She could not run or jump at school. She would fall asleep because she was really tired. The teachers stopped making her do physical activity” FG3

When asked about how asthma affected their children, parents immediately referred to limitations in their physical activity. About half of the participants recognized these limitations as an important source of emotional distress for their children:

– “My daughter is fine now, but she did use to get depressed when she could not do the same as others” FG1
– “She loves running, but she has to limit herself” FG2
– “She loves to walk, she wants to do that like my other child who participates in running competitions at school, so it is hard for her to not be able to do that” FG3

Four parents in two different focus groups brought up that their children felt embarrassed by these limitations or by having to use medication like their inhalers at school. They reported that their children were being teased at school because of their condition:

– *“He would say*: ‘*Mom! When you go to school, do not tell them that I cannot eat this or that, because the other kids make fun of me*’, *but I say*, ‘*what should I do? I have to tell them!*’*” FG1*
– “It affects them, because sometimes kids make fun of them” FG2
– “My kid would hide to use the inhaler, he was ashamed. He would hide the pump to avoid bringing it to school” FG2

#### Asthma Self-Management

Parents mentioned that one of the main challenges to controlling their children’s illness was identifying asthma triggers. Among the main triggers mentioned during the focus group interviews were smoke, chemicals, animals, dirt and dust, pollen, and stuffed animals. Cold weather and running or doing physical activity were also identified as risk factors for asthma attacks in their children. Once asthma triggers were identified, all parents reported changes in their routine in order to help their children avoid asthma attacks. These newly adopted self-management behaviors included changes in their household cleaning habits, such as switching to asthma-friendly cleaning products, changing bed sheets often, removing carpets, avoiding animal contact, and not smoking or grilling inside the house.

Coming to terms with the chronic nature of the disease had a direct impact in disease management, specifically ensuring that their children took their medication consistently. A common error acknowledged by parents in all the focus group discussions was that initially, they did not adhere to the consistent use of asthma medication or completely discontinued it when their children’s symptoms seemed to decrease:

– “Once a kid has asthmatic lungs, they will have it forever” FG2
– *“I used to do that, I will look at him doing well and wouldn*’*t give him the medicine and then he would get sick, but now I know” FG1*
– “The same way that I know that I have to feed them, I give him the medicine” FG1
– “*If my children do not take their medicine, they get very sick” FG3*

#### Asthma Education

Doctors and health personnel are key sources of information for parents. Most of the parents heard about the illness for the first time when their child was diagnosed with asthma, and they learned about it as they struggled to control their child’s illness. Participants shared their stories about the stressful learning process:

– *“It*’*s scary. I am always here with the medication. Sometimes I do not sleep. I am always worried” FG3*
– *“My two sons have had asthma since they were little, and [being] without education is difficult. After the [asthma] education, it*’*s different” FG3*

A valuable means for increasing asthma knowledge and asthma control was through asthma education. Those who mentioned receiving asthma education emphasized how important learning to identify asthma triggers was for disease management.

– “I have received asthma education, and what I have learned is to change cleaning products to avoid chemicals” FG2
– “Cleaning, all the things that you use for it, like sprays to make a room smell well, Chlorine” FG1
– “I used a lot chemical products when I cleaned. I learned how the dust sticks to the curtains. I cleaned, but not so often. I really liked when the promotoras came to my house and showed me what to do” FG3

## Discussion

This study provides important insights into the experience of Hispanic families who have children with asthma living in the US–Mexico border region. The population studied has considerable burdens to overcome due to a combination of challenges including, but not limited to, low socioeconomic status, language barriers, a diverse cultural background, and lack of knowledge of the health systems in the US. The results from the survey and the focus groups show that asthma significantly affects the quality of life of children and their families. Parents missed work and children missed school due to the child’s asthma, and despite the fact that most children have health insurance—mainly through Medicaid—many parents worried about the cost of asthma medication (21, 31). Therefore, it is not surprising that of the five dimensions measured by the CHSA, the child’s and family’s Emotional Health are the most affected by the disease, followed by the Child’s Physical Health. In contrast, the child’s and family’s activities were the least affected dimensions. This is consistent with focus group results, where the lack of asthma knowledge and asthma symptoms and limitations were consistently referred to as a source of frustration, stress, and anxiety for children and their families. On the other hand, parents and guardians reported having no problem changing certain behaviors, like cleaning habits, smoking, or having pets inside the house, in order to avoid asthma attacks. Additionally, the focus groups results highlighted the lack of asthma self-management skills among participants, diagnostic uncertainty, and some parents expressed that lack of medication adherence and compliance from their children was due to bullying at school. Other studies have reported similar behaviors in children with asthma (32).

Most of the interviewed parents and guardians had education levels of high school or less and had little knowledge of asthma before their child’s diagnosis. Less than half of the survey and focus groups participants had received asthma education in the past, despite having one or more children diagnosed with the disease. Focus group findings suggest that this lack of knowledge is one of the main challenges for parents of children with asthma when first faced with their children’s symptoms, as well as, an important barrier to asthma control. These results are consistent with Mosnaim et al. (33) and Vernon (34), and suggest that asthma education is needed in health-care, school, and community settings, in order to educate parents on ways to detect asthma in their children and avoid dangerous asthma attacks and potential emergency room visits. Timely identification of asthma triggers has proven to be an effective preventive measure (19, 35), and parents are willing to make changes in their homes as a result of the healthy homes training education they received.

Studies have shown that while asthma has an effect on the quality of life of children and their families, particularly in the emotional aspects, a wide dissemination of asthma management education in different settings can help control asthma symptoms and allow for better management of asthma (7). Recommendations to improve outreach to Hispanic families with children who have asthma include promoting asthma prevention at health fairs and community markets and establishing community coalitions with other groups already trusted by the community. Border initiatives such as the Program on Asthma Education and Research (7, 28) may alleviate the medication cost burden experienced by the parents, particularly when their children age out of Medicaid. Public health policy efforts may consider advocating for initiatives that provide free health-care referrals for asthmatic children, as well as, medication program enrollment at local hospitals. Findings from previous studies have shown that offering asthma management education to parents with diagnosed children may be an effective strategy to help them identify asthma triggers while also increasing treatment adherence in this population (7, 19, 20). Also, knowing how to effectively manage the disease reduces a great deal of the emotional burden experienced by children with asthma and their families. Further research is needed to determine what settings and type of health providers would be more effective in providing asthma preventive information to parents whose children have not yet been diagnosed.

Focus group interviews have increasingly been used in health research to explore cultural narratives, health beliefs, as well as the motivations behind health-related behaviors. They have become a valuable tool in pediatric research and have been used for program development and program evaluation (28, 30). In this study, the information obtained through the focus group discussions provide valuable insight to help interpret the survey findings while taking into account the particular characteristics of Hispanic families of children with asthma in Hidalgo County. Among the main limitations of the study are the small number of focus group participants and the use of convenience sampling, which limit the generalizability of the results. Despite these limitations, the findings from this study may be useful for those researchers and public health practitioners who work in tailoring asthma management programs for Hispanic residents in the South Texas–Mexico border.

## Ethics Statement

This study was carried out in accordance with the regulations of the Department of Health and Human Services for the protection of human research participants. All subjects gave written informed consent in accordance with the Declaration of Helsinki. The protocol was approved by the Institutional Review Board at Texas A&M University (IRB2015-0472D).

## Author Contributions

All authors contributed equally to this work.

## Conflict of Interest Statement

The authors declare that the research was conducted in the absence of any commercial or financial relationships that could be construed as a potential conflict of interest.
